# Unlocking near-whole-brain, layer-specific functional connectivity with 3D VAPER fMRI

**DOI:** 10.1162/imag_a_00140

**Published:** 2024-04-18

**Authors:** Yuhui Chai, A. Tyler Morgan, Hua Xie, Linqing Li, Laurentius Huber, Peter A. Bandettini, Bradley P. Sutton

**Affiliations:** Beckman Institute for Advanced Science and Technology, University of Illinois at Urbana-Champaign, Urbana, IL, United States; Section on Functional Imaging Methods, Laboratory of Brain and Cognition, National Institute of Mental Health, National Institutes of Health, Bethesda, MD, United States; Center for Neuroscience Research, Children’s National Hospital, Washington, DC, United States; Functional MRI Core, National Institute of Mental Health, National Institutes of Health, Bethesda, MD, United States

**Keywords:** connectivity, cortical layer, cortical depth, feedforward, feedback

## Abstract

Neuroscientific investigations at the cortical layer level not only enrich our knowledge of cortical micro-circuitry in vivo, but also help bridge the gap between macroscopic (e.g., conventional fMRI, behavior) and microscopic (e.g., extracellular recordings) measures of brain function. While laminar fMRI studies have extensively explored the evoked cortical response in multiple subsystems, the investigation of the laminar component of functional networks throughout the entire brain has been hindered due to constraints in high-resolution layer-fMRI imaging methodologies. Our study addresses this gap by introducing an innovative layer-specific 3D VAPER (integrated VASO and Perfusion contrast) technique in humans at 7 T, for achieving fMRI at high resolution (800 µm isotropic), high specificity (not biased toward unspecific vein signals as BOLD), high sensitivity (robust measurement at submillimeter resolution), high spatial accuracy (analysis in native fMRI space), near-whole-brain coverage (cerebellum not included), and eventually extending layer fMRI to more flexible connectivity-based experiment designs. To demonstrate its effectiveness, we collected 0.8-mm isotropic fMRI data during both resting-state and movie-watching scenarios, established a layer-specific functional connectivity analysis pipeline from individual to group levels, and explored the role of different cortical layers in maintaining functional networks. Our results revealed distinct layer-specific connectivity patterns within the default mode, somatomotor, and visual networks, as well as at the global hubness level. The cutting-edge technique and insights derived from our exploration into near-whole-brain layer-specific connectivity provide unparalleled understanding of the organization principles and underlying mechanisms governing communication between different brain regions.

## 1 Introduction

With increased availability of ultra-high field (≥7 T) human MRI scanners, functional MRI (fMRI) spatial resolution has been pushed to the sub-millimeter domain, making it possible to resolve functional activity and connectivity across cortical layers (Feinberg et al., 2018;L. Huber, Handwerker, et al., 2017;Weldon & Olman, 2021). Although true anatomical layers cannot be uniquely identified and measured with high-resolution fMRI, current sub-millimeter resolution does allow for depth-resolved measures of the hemodynamic response, providing roughly two to three independent observations over the depth of the cortex, at a similar spatial scale as the underlying three cortical “super” layers defined as infragranular, granular, and supragranular (Scheeringa et al., 2023). Cortical layers are believed to have distinct functional roles (Norris & Polimeni, 2019). Specifically, feed-forward, bottom-up activity predominantly terminates in the middle granular layer, while feedback, top-down activity mainly terminates in superficial and/or deeper layers (Felleman & Van Essen, 1991;Self et al., 2017). Both layer-specific activity (L. Huber, Handwerker, et al., 2017) and connectivity (L. Huber et al., 2021;Rockland, 2017;Yun et al., 2022) can be used to differentiate bottom-up and top-down cognitive processes and to define functional hierarchy among cortical areas (Stephan et al., 2017). While laminar fMRI studies have extensively explored the evoked cortical response in multiple subsystems, the investigation of the laminar component of functional networks across the entire brain has been hindered due to constraints in high-resolution layer-fMRI imaging methodologies. These constraints include the use of macro-vascular-contaminated sequence contrasts for functional measurement, poorly defined cortical layers roughly based on distortion mis-matched anatomical reference, limited brain coverage, and activity-based experiment design.

**Fig. 1.**
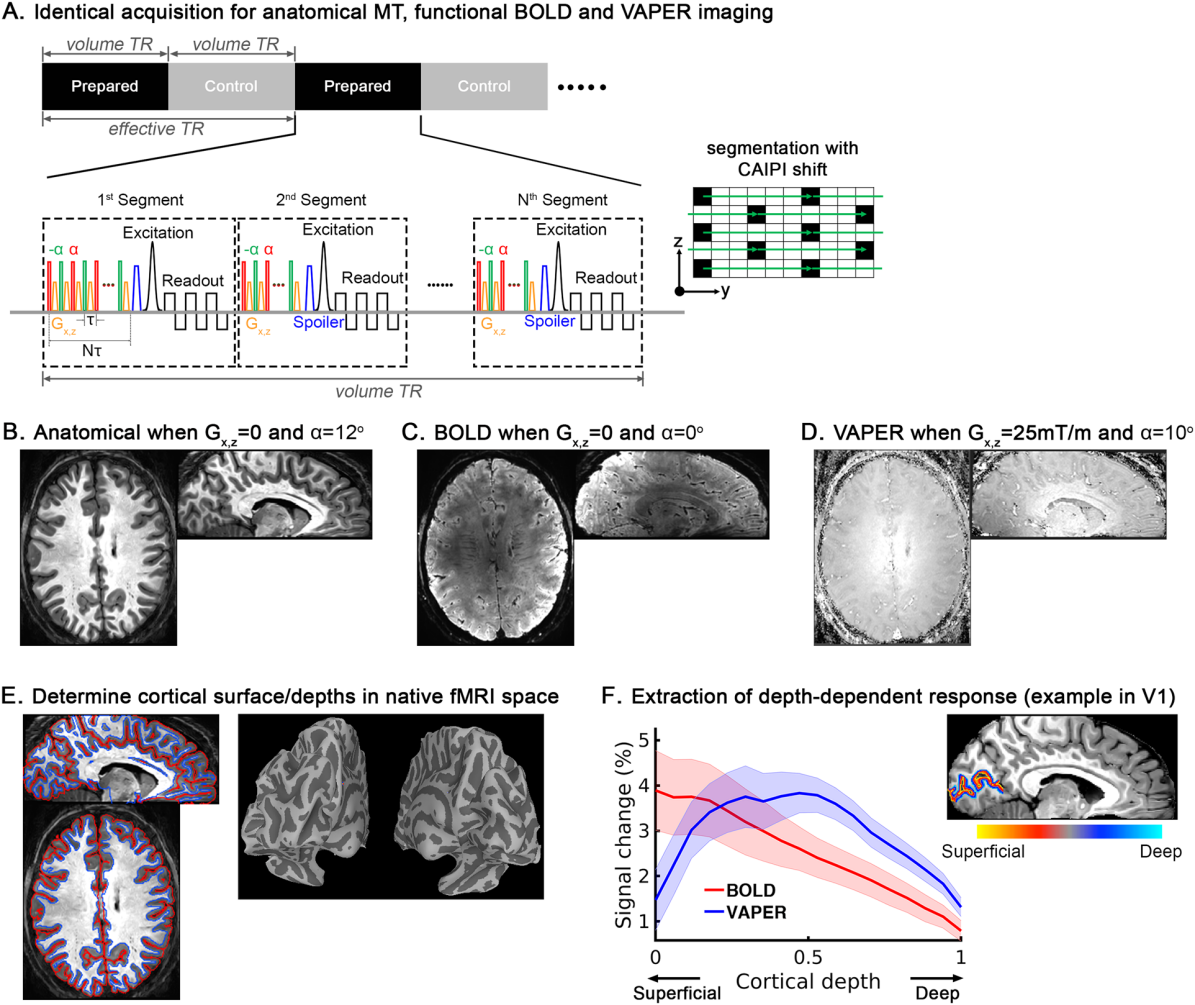
(A) Sequence design for functional and anatomical imaging, which utilizes an identical acquisition of shot-selective CAIPI 3D-EPI (in-plane segmentation with CAIPI shift). (B) Acquisition of anatomical MT-weighted images alternating between control and MT-prepared conditions. The gradients in the preparation module are switched off and power of RF pulses maximized up to the SAR limit to yield the optimal MT-weighted anatomical reference. (C & D) Acquisition of functional VAPER data alternating between blood-nulled module prepared and control conditions. Blood nulling is achieved by combining DANTE (Delay Alternating with Nutation for Tailored Excitation) pulse trains (turning on both gradients and RF pulses in the preparation) with 3D-EPI. In control volume, DANTE RF pulses are switched off to acquire the signal for BOLD correction. (E) Determination of cortical surface and depths in the native fMRI space based on anatomical MT-EPI images. (F) Extraction of laminar profile of BOLD (red curves) and VAPER (blue curves) response in V1 to visual checkerboard stimulus. The laminar profiles of visual response were averaged across eight subjects, and the shaded areas represent ± SEM (standard error of the mean) across subjects. As expected by the draining vein effect, BOLD response peaked in superficial cortical depths and decreased monotonically toward the white matter. In VAPER, such strong bias toward the pial surface was much weaker, and the response profile peaked closer to middle cortical depths.

Recent advancements in fMRI contrast and readout strategies offer potential solutions to these limitations. Studies in animal models have demonstrated that both cerebral blood volume (CBV) and cerebral blood flow (CBF) can be used to identify layer-dependent brain activation with superior spatial specificity compared to T_2_*-weighted BOLD (T. Jin & Kim, 2008). In previous work, we introduced a technique that integrates CBV and CBF into one contrast by using DANTE (Li et al., 2012) (Delay Alternating with Nutation for Tailored Excitation) pulse trains combined with 3D echo-planar imaging (EPI) to acquire an integrated blood volume and perfusion (VAPER) contrast (Chai et al., 2020). Additionally, we developed a magnetization transfer (MT) weighted anatomical EPI technique to facilitate determination of cortical depth in native fMRI space (Chai, Li, et al., 2021). This technique uses an identical acquisition with functional VAPER imaging and provides sufficient gray-white matter contrast to perform all analysis in the native fMRI space, eliminating the need for distortion correction and registration. In this study, we employed a segmented 3D-EPI with shot-selective controlled aliasing in parallel imaging (shot-selective CAIPI) sampling strategy (Norbeck et al., 2020;Poser et al., 2013;Stirnberg & Stöcker, 2020), as the acquisition for both functional VAPER and anatomical MT imaging (Fig. 1). Our DANTE and MT preparation can be evenly applied before each readout shot regardless of various EPI segmentation schemes and the acquisition length. Extending from a few slices toward whole-brain coverage, the EPI design remains unchanged, and the functional and anatomical contrast build up to steady state with the cumulative effect of more DANTE/MT preparations (one per each shot) with higher resolution and more shots. This tool allows for layer-specific functional and distortion-matched anatomical imaging with no limitations on acquisition length and segmentation factors, enabling layer-fMRI with any brain coverage (partial or whole brain) and spatial resolution (0.5–0.8 mm). To maintain a reasonable repetition time (TR) for fMRI connectivity measurement, we aimed for near-whole-brain 0.8-mm isotropic in this work.

This whole-brain VAPER-fMRI sequence tool enables noninvasive exploration of connectivity across various cortical depths in the human brain. In comparison to previous studies on layer fMRI connectivity (Deshpande et al., 2022;L. Huber et al., 2021;Koiso et al., 2023;Pais-Roldán et al., 2023;Yun et al., 2022), besides the more layer-specific VAPER measurement than BOLD, this advanced VAPER-fMRI sequence tool also provides several analytical benefits. First, in each individual, all analyses, including layer examinations, can be conducted in native fMRI space for maximum spatial and layer precision. Second, from individual to group level, laminar features are preserved through a cortical depth-specific surface registration. Third, a model-free k-means clustering analysis can be directly applied to each vertex, generating vertex-wise maps of laminar patterns. In previous layer fMRI studies, laminar analysis typically involves extracting laminar profiles at the ROI level (Chai, Liu, et al., 2021;L. Huber, Handwerker, et al., 2017;L. Huber et al., 2021) without creating voxel/vertex-wise laminar pattern maps. To generate such laminar feature maps, researchers have employed laminar profile modeling to distinguish different laminar patterns in each voxel unit (L. Huber et al., 2021). However, as the laminar profile model can be complex and regionally variable, a model-free k-means approach is more practical, particularly for whole-brain analysis. Additionally, we collected two submillimeter fMRI datasets with nearly whole-brain coverage (cerebellum not included) in this study: one during rest and the other during movie-watching. While a substantial portion of fMRI connectivity research has relied on resting-state data due to its minimal participant cooperation requirements (Biswal et al., 1995;Greicius et al., 2003), movie-watching has gained traction in human neuroimaging research as it is considered a more real-life context, improving participant arousal levels and reducing head motion during data acquisition (Finn & Bandettini, 2021;Tian et al., 2021). Our layer-specific functional connectivity analysis pipeline examines the involvement of different cortical layers in various brain areas for the maintenance of the brain networks in both datasets. Given that disruptions in cortical networks are often linked to psychiatric disorders, our study’s applicability could potentially pave the way for investigating the biological mechanisms and treatment strategies for these conditions.

## 2 Materials and Methods

### 2.1 fMRI participants

Twelve healthy volunteers (6 males, aged 21-31 years) gave informed consent to participate in this study under an NIH Combined Neuroscience Institutional Review Board approved protocol (93-M-0170, ClinicalTrials.gov identifier: NCT00001360). Eight of those participants underwent multiple fMRI sessions (2 hours in each session), resulting in a combined total of 36 sessions (72 hours). Details of each scan acquired can be found inTable S1.

### 2.2 Experiment paradigm

In order to illustrate the schematics of layer-fMRI connectivity using VAPER-fMRI, we conducted movie-watching and resting-state experiments. To mitigate head motion, we placed two inflatable air cushions in the empty space laterally between the participants’ head and the casing of the receive RF coil.

#### 2.2.1 Movie-watching

For the movie-watching experiment, we utilized an already established collection of five short video clips that was used in previous 7 T human connectome project (HCP) (https://www.humanconnectome.org/study/hcp-young-adult/article/first-release-of-7t-mr-image-data). In each session, we acquired five runs of watching the same movie, with each run lasting for 15 minutes. The near-whole-brain VAPER/MT-3D-EPI sequence was used for image acquisition.

#### 2.2.2 Resting state

During the resting-state experiment, we instructed the participants to keep their heads still and not fall asleep. In each session, we acquired two to three runs, with each run lasting for 20–30 minutes, resulting in a total duration of 1 hour.

### 2.3 Data acquisition

#### 2.3.1 Scanning setup

The experiments were performed on a Siemens MAGNETOM 7T scanner equipped with a Nova single-channel transmit/32-channel receive head coil. A 3^rd^order B0-shimming with three iterations was applied to the imaging region.

#### 2.3.2 VAPER-3D-EPI sequence for functional imaging

To acquire functional data, we introduced an integrated blood volume and perfusion (VAPER) contrast (Chai et al., 2020) acquired by combining the blood-suppression module of DANTE (Delay Alternating with Nutation for Tailored Excitation) pulse trains (Li et al., 2012) with 3D-EPI (Poser et al., 2010). The sequence was implemented to acquire fMRI images alternating between blood-signal-suppressed (DANTE prepared 3D-EPI) and blood-signal-augmented (original 3D-EPI as control) conditions (Fig. 1). Parameters of DANTE pulse train were as follows: pulse number in 1^st^/later segment = 120/18, pulse interval = 1 ms, pulse flip angle = 10°, gradient = 25.6 mT/m (applied along x and z directions simultaneously). Image acquisition parameters were as follows: TE = 20 ms, flip angle of water excitation = 19°, 96 slices (8 slices oversampling), imaging resolution = 0.8 × 0.8 × 0.84 mm^3^, matrix size = 176 × 220, partial Fourier of 7/8 in both phase encoding directions, and CAIPI 3 × 2 (k_z_shift 1) with 2 shots per k_z_-segment (Poser et al., 2013;Stirnberg & Stöcker, 2020) (For a systematic comparison of different CAIPI patterns applicable to near-whole-brain layer fMRI data acquisition, refer toKoiso et al. (2023)). This protocol results in a volume TR of 6.082 seconds, encompassing the preparation and acquisition period of the entire 3D volume. Due to the interleaved acquisition of DANTE-prepared and control volumes, the effective TR will be doubled, which is 12.164 seconds.

Through dynamically subtracting the signal in the blood-nulled condition from that in the control condition, VAPER contrast was generated to be sensitive to both cerebral-blood-volume (CBV) and cerebral-blood-flow (CBF) while BOLD weighting could be largely attenuated. To remove any remaining BOLD contamination, VAPER time series was further corrected through dynamical division by that of the control image to factor out the$\text{exp(}-TE/T_{2}^{\text{*}}\text{)}$term. In addition, the signal of the control condition is mainly determined by BOLD contrast, thus it can be treated as a conventional BOLD signal.

The laminar specificity of the whole-brain VAPER sequence has been verified using a basic visual checkerboard task (flickering rate of 10 Hz, 30 seconds ON / 30 seconds OFF, 30 minutes) as shown inFigure 1F.

#### 2.3.3 MT-3D-EPI sequence for anatomical reference

In the human brain, a relatively large fraction of macromolecular hydrogen protons (MP) (f~0.2–0.3) is found in white matter (WM), while this number is smaller in gray matter (GM) (f~0.1) (van Gelderen et al., 2017). Through magnetization transfer (MT) with water hydrogen protons (WP), MPs can dramatically affect the MRI signal and thus different MP fractions in GM and WM will lead to different MRI signal intensities. Here, we incorporated MT-weighted imaging with the fMRI acquisition technique to generate the anatomical image (Chai, Li, et al., 2021; Chai, Liu, 2021).

Its sequence design is almost identical to the functional VAPER imaging (Fig. 1). To switch from functional VAPER contrast to anatomical MT weighting, we turned off the gradients in the preparation and maximized the RF power of the preparation pulses (FA = 10–13°, minimal RF duration allowed under the SAR limit). We also acquired interleaved images between the MT-prepared and control conditions.

The MT-weighted anatomical image was generated as$\frac{S_{CTRL}-S_{MT}}{S_{MT}}$, where$S_{CTRL}$represents the image signal in the control condition, and$S_{MT}$represents the image signal of the MT-prepared condition. This combination approach extracts the MT-saturated signal and removes the T_2_* weighting associated with the EPI readout.

### 2.4 Data analysis

#### 2.4.1 Volume-based preprocessing

Functional MRI data underwent initial preprocessing in the volumetric space. After removing the first two volumes from each run, we performed motion correction on all functional VAPER and anatomical MT run imaging within a single session using SPM12 (Wellcome Trust Center for Neuroimaging, London, UK). Following that, we utilized the time series of fixed control and blood-nulled images from functional runs to compute the VAPER contrast, while the mean images of fixed control and MT-prepared conditions in the anatomical run generated the anatomical reference image. For both anatomical and functional data, we excluded time points from further analysis whenever the Euclidean norm of motion derivatives exceeded 0.4 mm or when outliers from the trend comprised at least 10% of image voxels.

To account for physiological and hardware related confounds, the voxel-wise time series of VAPER were regressed against the following variables: six head motion parameters and their derivatives, slow signal drift modeled with polynomials up to the fifth order, ventricular CSF signal, and voxel-wise local white matter regressors using the ANATICOR method (Jo et al., 2010). The VAPER time series were then normalized by each voxel’s mean signal across time, yielding residual VAPER time series for subsequent connectivity analysis.

#### 2.4.2 Cortical surface and depth reconstruction

At the individual level, we reconstructed cortical surface and depths on the MT-EPI images and projected the functional data onto the cortical surface at each cortical depth.

First, the MT-weighted EPI images were used to generate WM/GM segments and cortical surface (Fig. 1E) through the FreeSurfer program (Fischl, 2012). Then based on the automatically generated cortical surface, we calculated cortical depths using the equi-volume approach (Waehnert et al., 2014) with the Surface tools (Id et al., 2020;Wagstyl et al., 2018) (https://github.com/kwagstyl/surface_tools) and divided the cortex into 18 equi-volume layers. The choice to derive 18 layers allowed us to improve layer profile visualization and minimize partial voluming between neighboring voxels (L. Huber, Handwerker, et al., 2017;L. R. Huber et al., 2021). For a lower number of layers, multiple voxels with centroids across a wider range of cortical depths would have been binned into the same layer, which would have resulted in loss of resolution. In the whole context of this study, we used the term “laminar” or “layer” to indicate a measurement taken along the cortical depth, as opposed to the cytoarchitectonically defined cortical layers.

#### 2.4.3 Projection of the functional volumetric data onto the cortical surface mesh

To leverage the above cortical depths as a template for laminar functional data analysis, we projected volumetric data/maps onto the cortical surface of each depth. In order to minimize resolution loss during this projection, we up-sampled the functional volumetric data by a factor of 5 before projection and increased the vertex density (refinement iteration of 1) (Wang et al., 2022) of each cortical depth surface. After the volume-surface-projection, we applied surface smoothing (kernel FWHM = 3 mm) within each cortical depth.

#### 2.4.4 Cortical depth-specific surface registration for group analysis

To aggregate data from all individual for group analysis, we carried out a new cortical depth-specific surface registration, as illustrated inFigure 2. For each individual session, cortical depths were determined using anatomical MT-EPI images, and the preprocessed functional data were directly projected onto the cortical surface at each cortical depth without the need of distortion correction or anatomical-functional alignment. Subsequently, a surface registration matrix was computed from each individual to the group-averaged cortical surface. Then for each individual, this registration matrix was applied to each cortical depth separately, aligning the surfaces of each depth to the group-averaged surface of the corresponding depth (e.g., surface of depth 1 for each subject → group-averaged surface of depth 1, surface of depth 2 for each subject → group-averaged surface of depth 2, and so on). This depth/layer-specific surface registration blurred data only within cortical depth but not across depths, allowing the combination of data from different individuals at each cortical depth for group analysis without blurring any laminar signature.

**Fig. 2.**
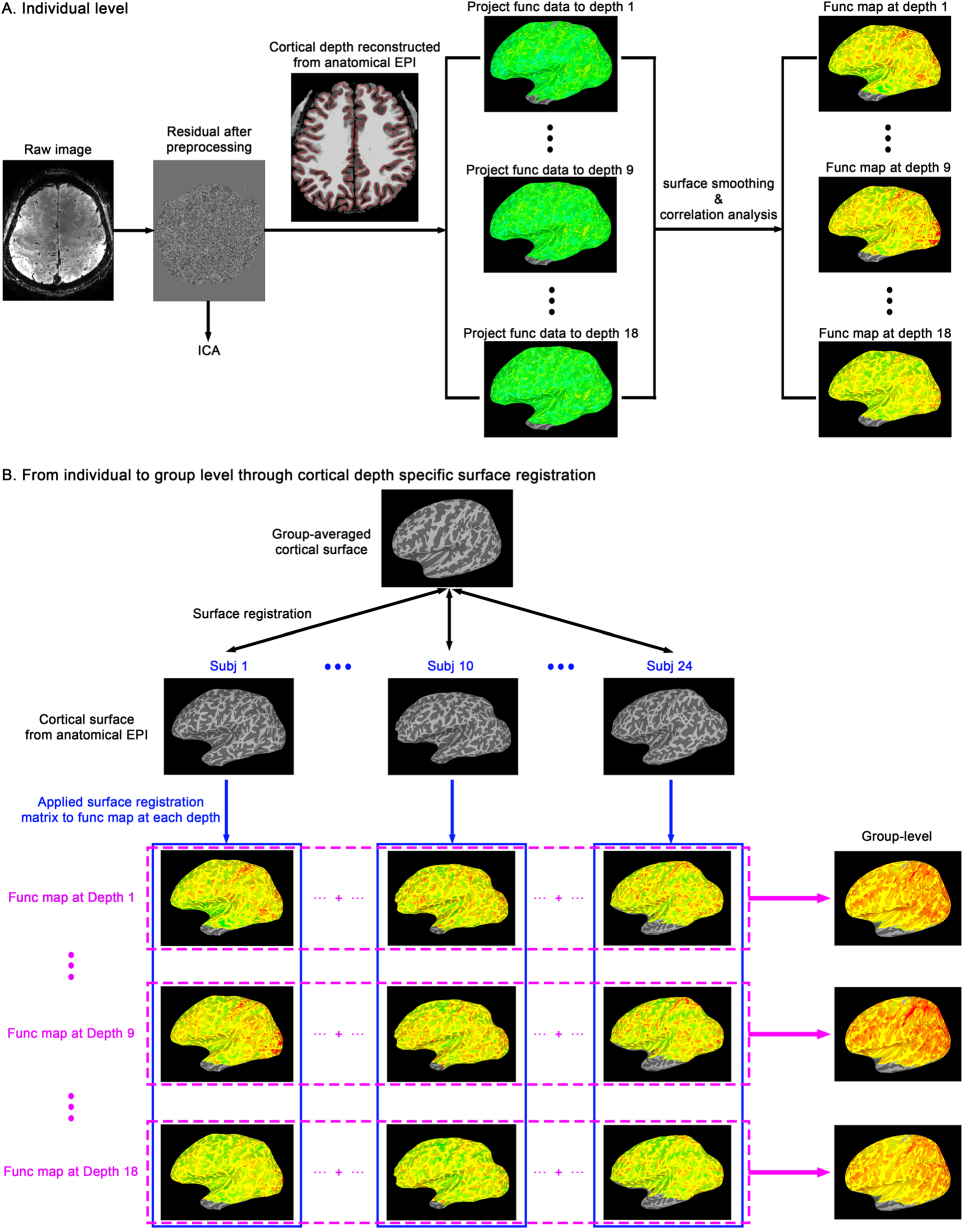
Flowchart illustrating the analysis process for projecting volumetric data to the cortical surface and implementing cortical depth/layer-specific surface registration. (A) functional data underwent preprocessing and ICA in the volumetric space. For each session, cortical depths were determined using MT-EPI images, and the preprocessed functional data were directly projected onto the cortical surface at each cortical depth, without the need for distortion correction or anatomical-functional alignment. (B) A surface registration matrix was computed from each individual to the group level. Then, this registration matrix was applied to each cortical depth separately, aligning the surfaces of each depth to the group-averaged surface of the corresponding depth. This depth/layer-specific surface registration blurred data only within cortical depth but not across depths. Following the depth-specific surface registration, the data of different individuals at each cortical depth can be combined for group analysis, without blurring any laminar signature.

#### 2.4.5 Network-level functional connectivity strength (FCS) for laminar analysis

For both resting-state and movie-watching data, we performed independent component analysis (ICA) using FSL program Melodic (Jenkinson et al., 2012) on each individual’s VAPER data in volumetric space. From all the automatically detected components (z-score > 2) in each session, we selected the components having the highest Dice similarity coefficient (Dice, 1945) with Yeo atlas (Thomas Yeo et al., 2011) to represent common functional networks, such as default mode, dorsal attention, and frontoparietal and somatomotor networks. To define the network regions for further analysis, we transformed the regions of default mode and somatomotor networks to the group-level cortical surface and then averaged them across all individuals as the mean network masks. For visual system analysis, we used the visual area template from the retinotopy atlas ofBenson et al. (2014), including V1, V2, V3 and higher-level visual areas (Benson et al., 2014). Within each network, we computed FCS of each vertex on the cortical surface, by correlating the time course of each vertex with all others within that network, transforming the correlations to Fisher Z-scores, and averaging the positive values. Based on the laminar profile of the FCS, different types of connectivity were distinguished for each vertex within each network.

To mitigate potential subjectivity in ICA statistic thresholding, we carried out a parallel analysis using the a priori default mode and somatomotor networks from Yeo atlas (Thomas Yeo et al., 2011).

#### 2.4.6 k-means clustering of FCS-based layer profile

Within each selected network, we applied k-means clustering (Goutte et al., 1999) to group areas according to the similarity of their FCS across 18 cortical depths. We first input vertex-wise FCS laminar profile in each network to the k-means clustering algorithm in MATLAB. This algorithm subsequently sorted network areas into two groups by maximizing within-cluster similarity and between-cluster dissimilarity, using correlation as a distance measure. This analysis was conducted on the group-averaged FCS map for each network. Considering our nominal resolution of 0.8 mm, the potential data blurring during either acquisition or analysis, and a typical cortical thickness of around 2.5 mm (Fischl & Dale, 2000), it is important to note that there are no more than 2–3 independent voxels within each voxel unit across cortical depths. Therefore, a cluster number of 2 is a reasonable choice, and more than that might lead to an overinterpretation of the data.

#### 2.4.7 Regions-of-interest (ROI) definition

We defined regions-of-interest (ROI) on cortical surface, barring the LGN, which was defined in volumetric space, for either seed-based connectivity analysis or extraction of functional connectivity laminar profiles. In the default mode network, we defined ROIs of the medial prefrontal cortex (mPFC), angular gyrus (ANG), and posterior cingulate cortex (PCC) based on the three main network nodes from ICA results. In the somatomotor network, we defined ROIs of Brodmann areas 1 (BA1), 3b (BA3b), 4a (BA4a), and 4p (BA4p), based on the Brodmann area maps from FreeSurfer reconstructed cortical surface results. For visual network analysis, we defined ROI of the lateral geniculate nucleus (LGN) based on the functional activated region in LGN, and the areas of V1, V2, and V3 according to the retinotopy atlas developed byBenson et al. (2014).

#### 2.4.8 ROI seed-based connectivity analysis

From each seed, we extracted the time series of all vertices/voxels within the ROI. Next, for every other vertex within the network that the seed belongs to, its correlations to all seed voxels were computed. We then averaged all positive correlations as the FCS at that vertex. The connectivity laminar profiles obtained from these analyses were used to classify the dominance of feedforward versus feedback connections. In a simplified canonical layer model (Lawrence et al., 2017;Markov et al., 2014;Self et al., 2017;Shipp, 2005), feedforward connections transmit information from lower to higher-level areas (bottom-up), while feedback connections relay information from higher- to lower-order areas (top-down). By examining the laminar differences in FCS between areas, we can determine the types of feedforward or feedback connections present.

#### 2.4.9 Regressing out visual response before connectivity analysis in visual cortex

For the connectivity assessment in movie-watching scans, to avoid the interference from the synchronized activity response induced by the low-level visual stimulus, the mean time course of the visual response was added as a regressor-of-no-interest and regressed out from the time series of all voxels in the visual system (including LGN and visual cortex). In this way, the resulting time series used for visual connectivity analysis would not be dominated by the mean visual response. For comparative purposes, the laminar profiles of connectivity measures were investigated in movie-watching scans also without regression of the visual response (Fig. S5).

#### 2.4.10 Global hubness

The global hubness, also known as global connectivity, refers to the FCS of each voxel/vertex with the entire cerebral cortex. It was computed by correlating the time series of every vertex on the cortical surface with all other vertices of the entire cerebral cortex (Kraus et al., 2020). To mitigate the computation burden arising from the extensive number of vertices, we parcellated the whole gray matter evenly into 1000 nodes using LAYNII program LN2_COLUMNS (L. R. Huber et al., 2021). For each node, a representative time series was extracted by averaging across all vertices within that node. Then, we computed the Pearson correlations of each vertex on the cortical surface at each cortical depth with all 1000 nodes and transformed them to Fisher Z-scores. The mean value of all positive correlations was calculated as the FCS of each vertex with the whole brain, as known as hubness.

#### 2.4.11 Extraction of laminar profiles for each network and whole-brain connectivity strength

To calculate connectivity profiles across laminar depths, we included all vertices within each network without applying any statistical thresholding. For the purpose of determining the laminar connectivity types, we normalized the laminar profile for each individual to the 0–1 range for highlighting the profile shape rather than its absolute values, averaged these normalized profiles across all sessions to pinpoint the cortical depth at which the peak connectivity strength resides. Please note that this normalization was for laminar profile visualization purpose only. The above k-means analysis pipeline was applied to the original laminar profiles without normalization.

#### 2.4.12 Test-retest reliability and statistical analysis

To assess the similarity of k-mean clustering outcomes, we used the Dice similarity coefficient (Bowring et al., 2019;Dice, 1945) (detailed in theSupplementary Materials) to compare the parcellation patterns obtained across different sub-groups of datasets. (1)**Comparing between movie-watching and resting-state datasets:**For each network’s k-means parcellation maps, we computed the Dice similarity coefficient between those from the movie-watching and resting-state datasets. (2)**Reliability of split-half analysis:**In both the movie-watching and resting-state datasets, we divided the individuals randomly into two equally sized subsets, and repeated this process 50 times. Within each network area, we applied the k-means clustering algorithm on the mean connectivity maps of each subset and obtained 50 pairs of parcellation results. Then, we computed the Dice similarity coefficient between each pair of parcellation patterns for each network, separately for movie-watching and resting-state datasets.

To statistically assess the aforementioned reliability measurements against the null hypothesis, we generated 50 pairs of random patterns within each network by randomly assigning vertices as either k = 1 (indicating laminar profiles peaking at superficial layers) or k = 2 (representing laminar profiles peaking at middle layers). The Dice similarity coefficients were computed for each pair of these random patterns. Subsequently, we performed two-sample t-tests to compare the Dice coefficients acquired from the actual measurements with those derived from the random patterns.

## 3 Results

Functional VAPER data acquired from each individual session enabled the identification of common networks through Independent Component Analysis (ICA) across both movie-watching and resting-state scenarios, as depicted inFigure 3. Within these networks, we computed the functional connectivity strength (FCS) of each vertex on the cortical surface, by correlating the time course of each vertex with all others within that particular network. The resultant FCS maps of each network were subsequently transformed from the individual to the group level via a cortical depth-specific surface registration process (Fig. 2, detailed in Methods). In the following sections, we utilized the group-level FCS in default mode, somatomotor, and visual networks, as well as the global hubness as representative examples to demonstrate the capabilities of VAPER-fMRI in conducting layer-specific functional connectivity research. Within each of these networks, and for the global hubness map, we applied a binary classification to segregate areas by their connection types, further examining the laminar characteristics of each network node.

**Fig. 3.**
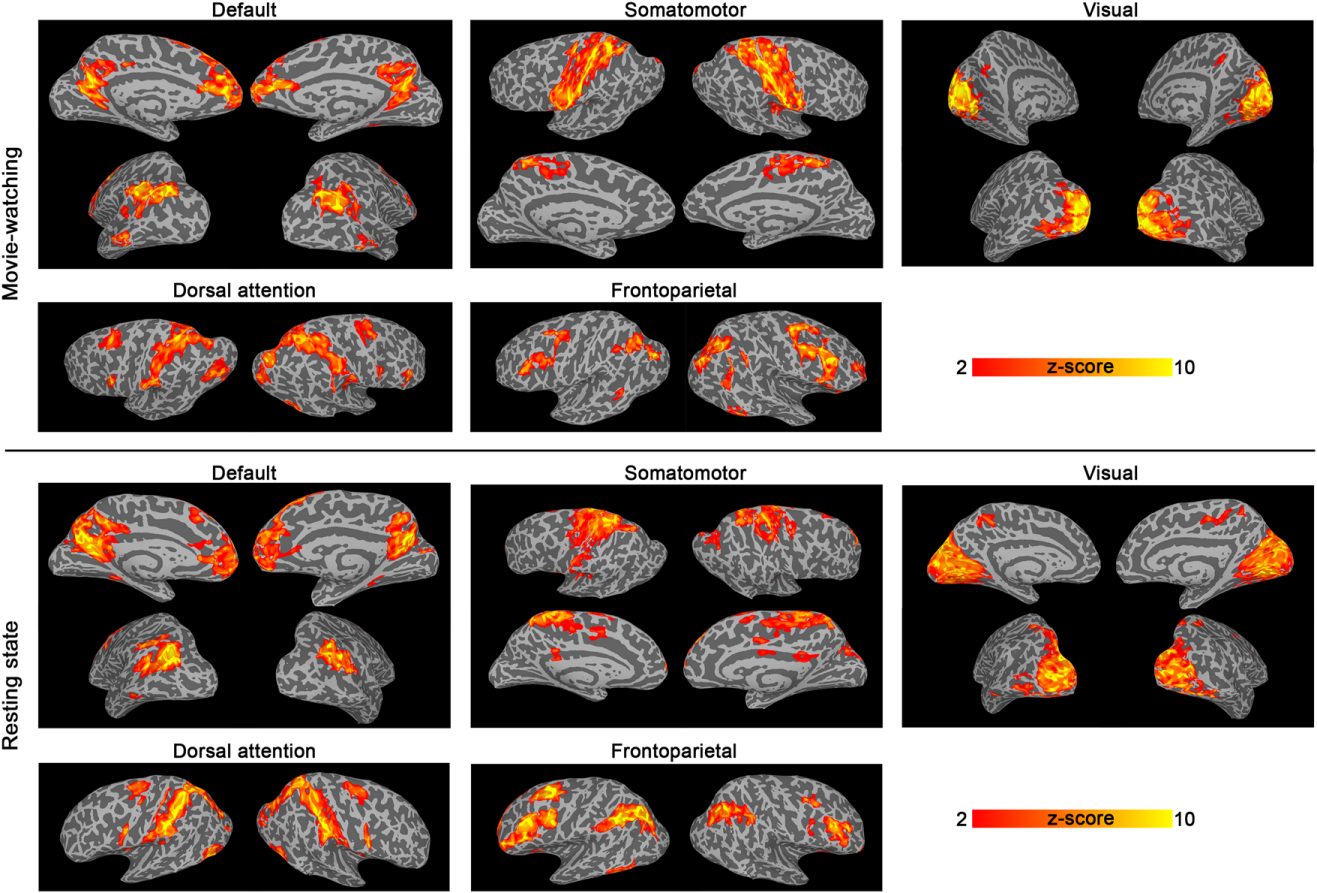
Movie-watching and resting-state networks with near-whole-brain high-resolution (0.8 mm isotropic) VAPER-fMRI. The upper panel displays data from one participant during the movie-watching experiment, while the lower panel presents data from another participant during the resting-state experiment. The underlays depict the cortical surface reconstructed from anatomical MT-EPI images, while the overlays indicate the networks detected using ICA based on VAPER data. This map suggests the sensitivity of submillimeter whole-brain VAPER-fMRI is sufficient for connectivity measurements.

### 3.1 Default mode network

Within the group-averaged default mode network (DMN), we utilized k-means clustering to identify areas with similar laminar profile of the FCS. Based on the cluster-mean layer profiles, we distinguished two types of connectivity here: one with peak FCS at the superficial cortical depths, indicating a feedback type of connectivity, and the other with peak FCS at middle layers, suggesting a feedforward type of connectivity.

**Fig. 4.**
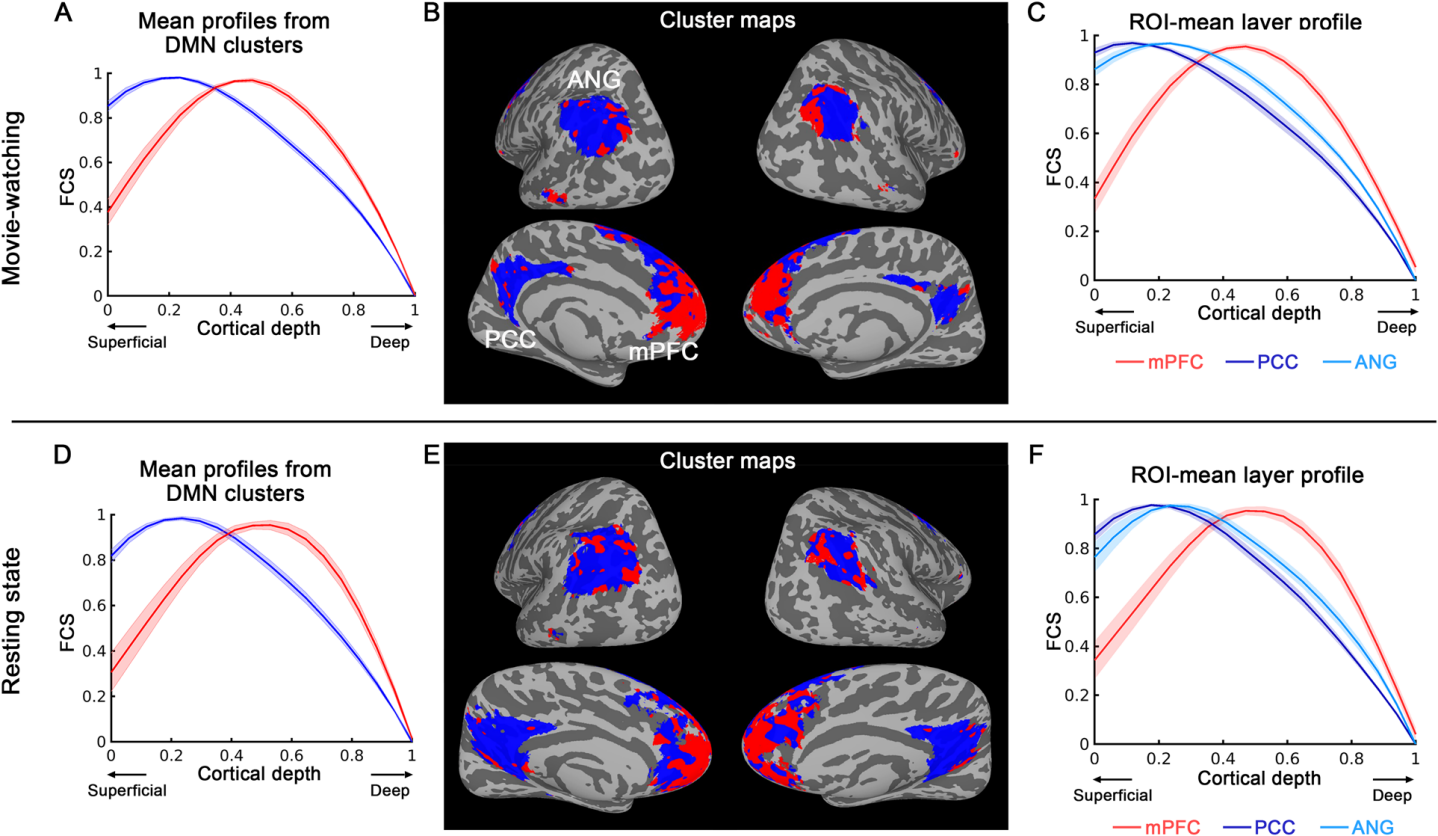
Laminar profile-based parcellation of default mode network (DMN) using k-means clustering at the group level (N = 24 for movie-watching and N = 12 for resting state). The upper panel showcases the results from the movie-watching experiment, while the lower panel presents results from the resting-state experiment. The DMN network region was individually defined using ICA and then averaged at group level. k = 2 was employed, and the mean functional connectivity strength (FCS) for each cluster was plotted as a function of cortical depth, as shown in (A) and (D). In (B) and (E), red regions represent FCS peaking at middle cortical depths, while the blue regions indicate FCS peaking at superficial cortical depths. (C) and (F) depict the mean laminar profiles of FCS within three main nodes of the DMN. The medial prefrontal cortex (mPFC) primarily exhibits feedforward-driven feature, with FCS peaking at middle cortical depths. Conversely, the inputs to angular gyrus (ANG) and posterior cingulate (PCC) are predominantly feedback-driven, with FCS peaking at superficial cortical depths. The shaded areas represent ± SEM (standard error of the mean) across sessions. For visualization purpose, the laminar profiles of FCS were normalized to the range of 0 to 1, while this normalization was not implemented in the k-means analysis pipeline.

Figure 4presents the clustering results of different laminar patterns within DMN at group level. The upper panel depicts results from the movie-watching dataset (N = 24), while the lower panel represents results from the resting-state dataset (N = 12). The DMN network region was defined via ICA in each individual session and then averaged at the group level. We employed a k-means clustering with k = 2 and plotted the mean FCS for each cluster as a function of cortical depth (Fig. 4Aand4D). InFigure 4Band4E, areas in red display FCS peaking at middle cortical depths, whereas those in blue indicate FCS peaking at superficial cortical depths. Our clustering analysis highlighted that feedforward connectivity type (illustrated in red, with a peak FCS at middle layers) is primarily localized in the medial prefrontal cortex (mPFC).Figure 4Cand4Fdepict the mean laminar connectivity profiles within three major nodes of the DMN: mPFC, posterior cingulate cortex (PCC), and angular gyrus (ANG). The mPFC exhibits a feedforward-driven feature, with FCS values peaking at middle cortical depths. In contrast, the inputs to the ANG and PCC exhibit predominantly feedback-driven connectivity, with FCS values peaking at superficial cortical depths. These clustering patterns were found to be similar across the movie-watching (upper panel ofFig. 4) and resting-state (lower panel ofFig. 4) datasets, indicated by a Dice similarity coefficient of 0.80 (compared to 0.5 ± 0.002 for random patterns in the same area, detailed statistics inTable S2) for the k-means clustering map.

In addition, we performed a parallel analysis within the DMN defined by the Yeo atlas (Thomas Yeo et al., 2011), and obtained consistent results as shown inFigure S1.

To further clarify the feedforward and feedback relationships between each pair of DMN nodes, we conducted a seed-based connectivity analysis by placing seeds in different DMN node. For every instance, we used one of the three nodes (mPFC, ANG, PCC) as the seed ROI and computed its correlation with the remaining DMN areas at each cortical depth. When using PCC or ANG as the seed, its correlation with the mPFC peaks at middle cortical depths (Fig. S2, left column). Conversely, when using mPFC as the seed, the correlation to PCC and ANG peaks at mainly superficial cortical depths (Fig. S2, right column). It suggests that mPFC receives feedforward inputs from ANG and PCC while ANG and PCC receive feedback inputs from mPFC. These results were observed in both the movie-watching (upper panel ofFig. S2) and resting-state (lower panel ofFig. S2) datasets.

Overall, these findings provide compelling evidence for distinct laminar connectivity profiles across DMN sub-network nodes and show consistent results across different datasets and network definitions.

### 3.2 Somatomotor network

Given that the granular layer, located at middle cortical depth, is nearly absent in the motor cortex (Dumoulin, 2017), the canonical feedforward/feedback model cannot be universally applied across the entire somatomotor network. Nevertheless, we are still able to identity different connection types within this network that aligned well with the findings from previous layer fMRI research in this area.

**Fig. 5.**
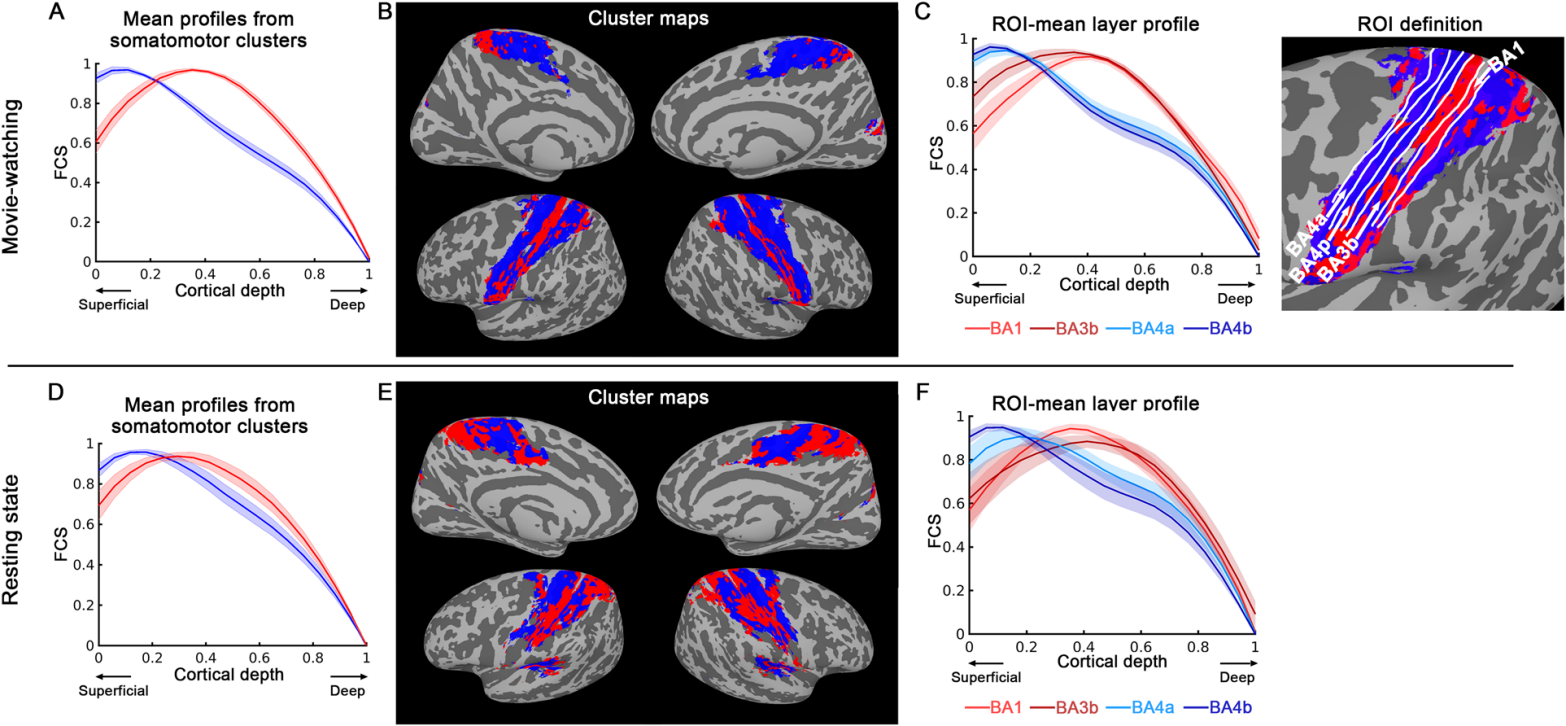
Laminar profile-based parcellation of somatomotor network using k-means clustering at the group level (N = 24 for movie-watching and N = 12 for resting state). The upper panel displays the results from the movie-watching experiment, while the lower panel presents results from the resting-state experiment. The somatomotor network region was individually defined using ICA and then averaged at group level. k = 2 was employed, and the mean FCS for each cluster was plotted as a function of cortical depth, as shown in (A) and (D). In (B) and (E), red regions have a laminar profile of FCS peaking at middle cortical depths. Conversely, the blue regions indicate a laminar profile peaking at superficial and slightly at deep cortical depths. (C) and (F) depict the mean laminar profiles of FCS within four regions-of-interest (ROI) of the somatomotor network. The laminar profile of BA1 and BA3b exhibit peak FCS at middle cortical depths, while that in BA4a and BA4p show a double-bump feature, with FCS peaks at both superficial and deep cortical depths. The shaded areas represent ± SEM across sessions. ROI boundaries are delineated by white lines on an exemplary k-means map.

Figure 5illustrates the clustering results of different laminar patterns within the somatomotor network at the group level. The upper panel represents the results from the movie-watching dataset (N = 24), while the lower panel represents the results from the resting-state dataset (N = 12). We individually identified the somatomotor network region using ICA, subsequently averaging it at the group level. We applied k-mean clustering with k = 2, and plotted the mean FCS for each cluster as a function of cortical depth, as shown inFigure 5Aand5D. InFigure 5Band5E, red regions indicate FCS peaking at middle cortical depths, while blue regions indicate FCS peaking at superficial and slightly deep cortical depths.

The cluster analysis revealed a distinct pattern within the somatomotor network. Specifically, somatosensory areas BA1 and BA3b showed peak FCS at middle cortical depths, whereas BA4a and BA4p (motor cortex) exhibited laminar profiles showing a weak double-bump features, as depicted inFigure 5Cand5F. This pattern was found to be similar across the movie-watching (upper panel ofFig. 5) and resting-state (lower panel ofFig. 5) datasets, indicated by a Dice similarity coefficient of 0.65 (compared to 0.5 ± 0.001 for random patterns in the same area, detailed statistics inTable S2) for the k-means clustering map.

Furthermore, we performed a similar analysis within the somatomotor network defined by the Yeo atlas (Thomas Yeo et al., 2011), and the results were consistent, as shown inFigure S3.

Collectively, these findings provide robust evidence for distinct laminar connectivity profiles within the somatomotor network and show consistent results across different datasets and network definitions.

### 3.3 Visual network

We investigated the laminar features of the visual system’s functional connectivity strength, employing an approach akin to the ones used for the aforementioned DMN and somatomotor network. Through the analysis of connectivity layer profiles across vertex pairs, we performed a k-means clustering to parcellate the visual cortex into two clusters. The results, illustrated inFigure 6Aand6B, show that low-level visual areas like V1 and V2 have peak FCS at superficial cortical depths, indicating a more feedback role when considering the visual system as a whole. In contrast, high-level visual areas show stronger FCS at mid-to-deep layers, suggesting a more feedforward-driven role in this system. This pattern was demonstrated through a reliability test, with a Dice similarity coefficient of 0.63 ± 0.19 (significantly different compared to 0.5 ± 0.001 for random patterns in the same area, detailed statistics inTable S2).

**Fig. 6.**
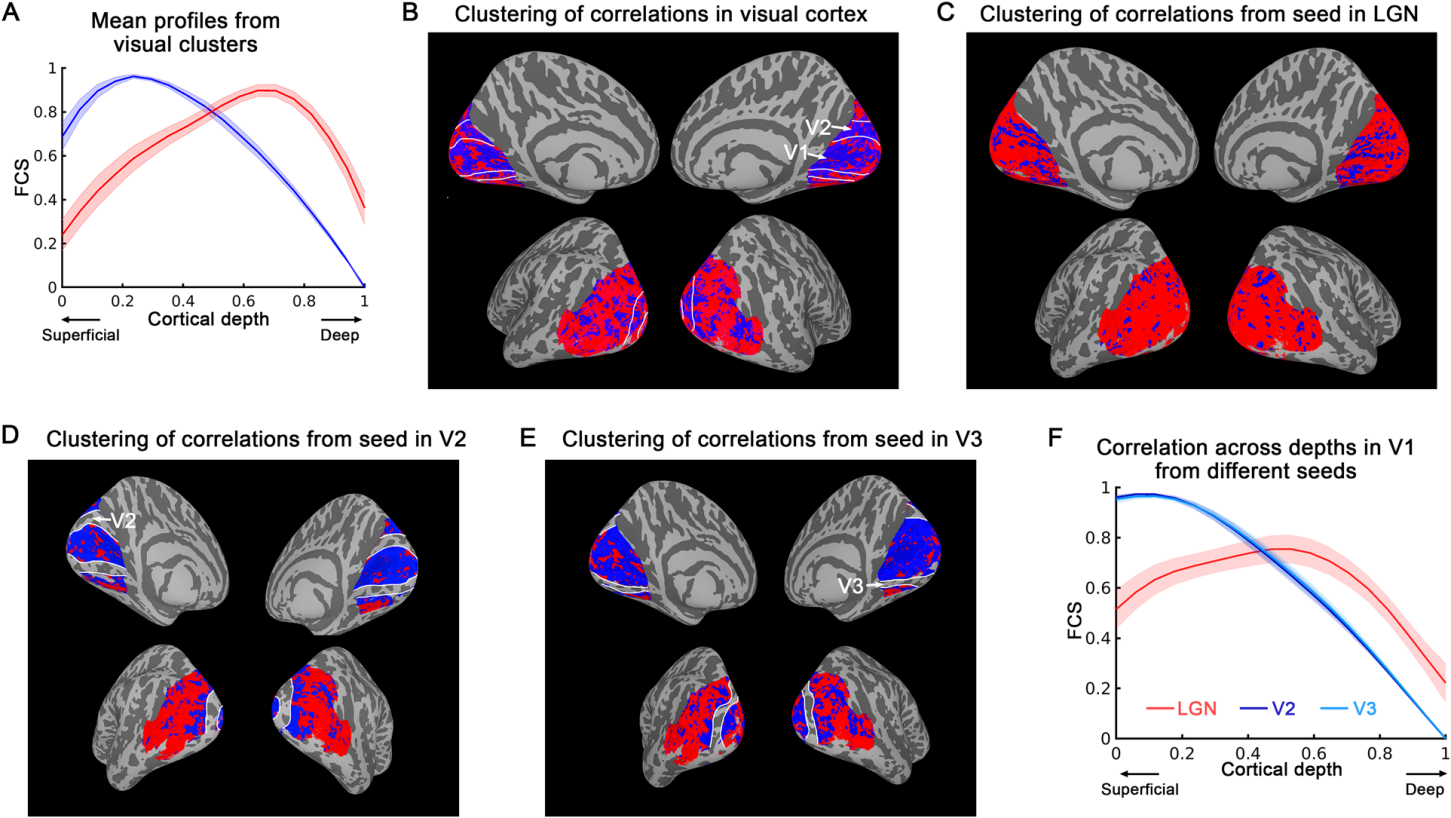
Laminar profile-based parcellation of visual system using k-means clustering at the group level (N = 24, movie-watching). The regions and sub-regions of the visual cortex were defined based on the retinotopy atlas ofBenson et al. (2014). (A) k = 2 was employed, and the mean FCS for each cluster was plotted as a function of cortical depth. (B) Parcellation of the visual cortex based on the laminar profiles of FCS. (C) Parcellation of the visual cortex based on the correlation with the seed in LGN. (D) Parcellation of the visual cortex (excluding V2 as it is used as a seed area) based on the correlation with the seed in V2. (E) Parcellation of the visual cortex (excluding V3 as it is used as a seed region) based on the correlation with the seed in V3. (F) The mean layer profiles of correlations in V1 with seeds in LGN, V2, and V3. When using the seed in LGN, V1 exhibits peak correlation at middle cortical depths. However, with seeds in V2 and V3, the peak correlations in V1 shift towards the superficial cortical depths. The shaded areas represent ± SEM across sessions. V1/2/3 boundaries are delineated by white lines on k-means maps.

To demonstrate how layer-specific connectivity can be used to study the hierarchical relationship between brain areas, we examined the evolution of laminar connectivity features by placing seeds at different hierarchical levels of the early visual system (LGN -> V1 -> V2 -> V3). From each seed region, we extracted the mean time course and computed a seed-based correlation map of the visual cortex. Initially, the seed was placed in LGN, and the results inFigure 6Cindicate that all visual areas exhibit the strongest correlation at middle to deep layers, suggesting that the entire visual cortex receives feedforward inputs from the LGN. Subsequently, we moved the seeds to V2 or V3, resulting in a shift in the laminar profiles of V1 and the areas below the seed regions in the visual hierarchy.Figure 6Dand6Edemonstrates that these regions now exhibit peak correlation towards superficial cortical depths, suggesting that they receive feedback connections from the seed regions. In contrast, higher-level visual areas still exhibit stronger correlations in middle-deep cortical depths, indicating a predominantly feedforward-driven connectivity pattern. To compare the laminar profiles of correlations based on different seeds, we extracted the correlation values in V1 across different cortical depths, as shown inFigure 6F. The results indicate that V1 receives feedforward connectivity when the seed is placed in LGN, while its connectivity becomes more feedback-driven when the seeds are moved to V2 and V3. This feature was also observed in the resting state, as illustrated inFigure S5.

### 3.4 Global hubness

Lastly, we examined the laminar pattern of the global hubness map. For each vertex, we computed its hubness as the mean positive correlation with the entire cerebral cortex, considering all cortical depths. Based on the hubness values across cortical depths, we parcellated the entire cerebral cortex into two clusters. One cluster exhibited the highest hubness strength at middle cortical depths, as represented by the red curve and cluster inFigure 7. In contrast, the other cluster showed the highest hubness strength toward the cortical surface, represented by the blue curve and cluster inFigure 7. The cluster with the maximum hubness at middle cortical depths was predominantly located in the prefrontal cortex (PFC), indicating that the PFC occupies a relatively high level within the whole-brain hierarchy system. This pattern was found to be similar across the movie-watching (upper panel ofFig. 7) and resting-state datasets (lower panel ofFig. 7), indicated by a Dice similarity coefficient of 0.78 (compared to 0.5 ± 0.001 for random patterns in the same area, detailed statistics inTable S2) for the k-means clustering map.

**Fig. 7.**
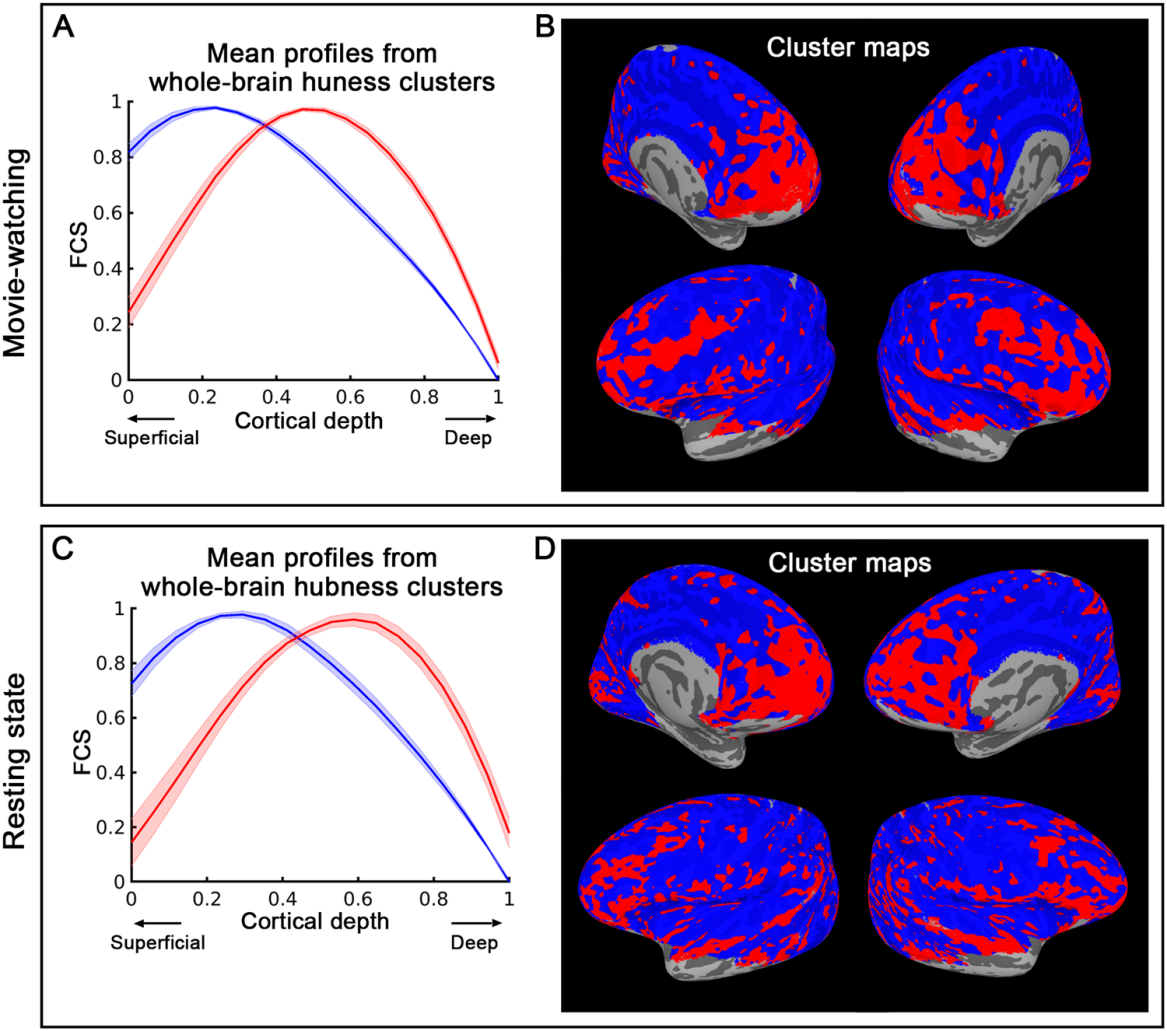
Laminar profile-based parcellation of the global hubness (global connectivity strength) map using k-means clustering at the group level (N = 24 for movie-watching and N = 12 for resting state). The upper panel displays the results from the movie-watching experiment, while the lower panel presents results from the resting-state experiment. k = 2 was employed, and the mean FCS for each cluster was plotted as a function of cortical depth, as shown in (A) and (C). In (B) and (D), red regions own a laminar profile of hubness peaking at middle cortical depths, while blue regions indicate the strongest hubness at superficial cortical depths.

## 4 Discussion

In this study, we illustrated how near-whole-brain layer-dependent VAPER-fMRI can be used for an innovative series of connectivity analyses, by generating multiple examples of analysis procedures and networks. We outlined several aspects in which our study advanced the field of layer fMRI and fMRI connectivity. We also acknowledge certain limitations and potential avenues for future improvement.

### 4.1 Technical advancements of the present work

#### 4.1.1 Near-whole-brain layer fMRI sequence tool for layer-specific connectivity studies

Our study adopted the advanced layer-specific fMRI tool, VAPER/MT-3D-EPI, that enables identical acquisition for both anatomical and functional imaging. There have been a handful of whole-brain layer fMRI connectivity studies (Deshpande et al., 2022;Pais-Roldán et al., 2023;Yun et al., 2022). Some of them are using BOLD-fMRI, whose signal is particularly susceptible to draining vein artifacts thus biased towards the cortical surface. As such, the interpretation of laminar effect using BOLD can be challenging if the effect of interest shows a steady increase towards the pial surface. Some aspects of this bias might be mitigated through de-veining model analysis like phase regression (Stanley et al., 2021). To more effectively overcome this limitation, a whole-brain layer-specific VASO-fMRI sequence has been developed for connectivity measurements in humans (L. Huber et al., 2021;Koiso et al., 2022). This VASO approach has successfully revealed distinct laminar features of feedforward and feedback-type connections across various networks. Our VAPER-fMRI utilizes an intravascular contrast similar to that in VASO, whose signal exhibits a superior laminar specificity compared to BOLD (Fig. 1). Additionally, even though the idea of distortion-matched anatomical imaging for fMRI and fMRI analysis in native EPI space has been explored in multiple studies previously (Brammer et al., 1997;Chai, Li, et al., 2021;Grabner et al., 2014;Kashyap et al., 2017;Renvall et al., 2016;Sanchez Panchuelo et al., 2021;van der Zwaag et al., 2018), the majority layer fMRI researches including layer fMRI connectivity (Deshpande et al., 2022;Pais-Roldán et al., 2023;Pfaffenrot et al., 2021;Scheeringa et al., 2023;Stanley et al., 2021) still predominantly rely on an anatomical image with different contrasts or acquisitions than functional ones. This necessitates co-registration between functional and anatomical datasets, which can blur functional data and is imprecise at the cortical layer level. By utilizing an identical scanning technique for both anatomical and functional data acquisition, our study allows for direct analysis within the native fMRI space, setting a new standard for future layer-specific fMRI connectivity research.

#### 4.1.2 Cortical depth-specific surface registration: necessary for group-level laminar pattern mapping

From an analytical standpoint, our work pioneers a new approach for group-level layer fMRI connectivity mapping. Due to the low signal-to-noise ratio (SNR) at high resolution, repeated averaging is necessary to attain functional statistical significance. Layer fMRI has previously struggled with the issue of spatial averaging across subjects. Spatially align and averaging across subjects for group-level analysis is challenging for layer fMRI due to the variable curvature of the cortical ribbon among individuals (Zilles et al., 1997). Moreover, conventional volume registration methods often blur information across cortical depths. To the best of our knowledge, there has been no successful group-level human brain map of whole-brain layer fMRI patterns. Until now, layer-specific fMRI research has merely averaged or compared the 1D laminar profile plots across subjects. Our study overcomes those obstacles by introducing an innovative cortical depth-specific surface registration method. This novel approach resolves the inherent challenges of layer fMRI and promises more accurate data analysis and interpretation. For the first time, we have constructed a group-level brain map of fMRI connectivity layer patterns, marking a significant milestone in understanding the intricate architecture of brain networks.

#### 4.1.3 Benefits of surface-based layer fMRI analysis over volumetric approaches

Implementing layer-specific fMRI in surface space delivers additional benefits, particularly for whole-brain layer connectivity analysis. High-resolution layer fMRI dramatically increases data size relative to conventional fMRI. The computational load for layer fMRI connectivity analysis can be significantly reduced by projecting volumetric data into surface space. A brief note on our data size for each functional time point: the volumetric data comprise 176 frequency-encode × 220 phase-encode × 96 slices × 4^3^upsampling factor (necessary to grow 18 layers in the volumetric space) = 237,895,680 voxels, whereas the surface data contain ~ 1213419 vertices (after refinement iteration) × 18 cortical depths = 21,841,542 vertices. The data size of the volume-based data size is more than 10 times of the surface-based data size, potentially leading to memory shortage issues in certain analysis programs.

Surface-based analysis also has a natural advantage. Since the cortical depth is derived from a family of surfaces with the same mesh or grid topology, there is an innate vertex correspondence between the same vertices (mesh nodes) at different cortical depths (Polimeni et al., 2017), a property which would be lost for voxels at different layers in volume space. This vertex correspondence makes it straightforward to generate a cortical depth profile for each vertex, further allowing for vertex-wise laminar profile analysis, such as the k-means clustering in this study. Ultimately, this enables the use of a surface map to represent the three-dimensional laminar feature of the cortex.

Most crucially, surface-based data analysis facilitates cortical depth-specific surface registration from individual to group level, a feat currently not possible with volume-based processing.

### 4.2 Layer-specific connectivity patterns across different brain networks

Our research uncovers distinct layer-specific connectivity patterns within various networks such as the default mode, somatomotor, and visual networks, as well as on the global hubness level. Those laminar patterns within each network look similar across resting-state and movie-watching. Movie viewing may have influence on the whole-brain functional network architecture; however, previous work has also shown that the connectivity over the resting-state and movie-watching sessions exhibited similar patterns (r = 0.8) (Cole et al., 2014;Demirtaş et al., 2019). Moreover, task-specific connectivity changes are likely to be smaller as compared to differences in connectivity pattern (Gratton et al., 2018), as our group averaging results may mask meaningful task-specific connectivity patterns. The current work lays the foundation, and future work is needed to further investigate the task-specific layer connectivity patterns.

#### 4.2.1 Default mode network: differing dominance of feedforward and feedback mechanisms across nodes

Within the DMN, we identified two distinct types of connectivity based on the laminar feature: feedback and feedforward. The mPFC, a key hub within the DMN, predominantly displayed a feedforward-driven pattern with a connectivity strength peak at middle cortical depths. Conversely, the inputs to the PCC and ANG demonstrated primarily feedback-driven connectivity with FCS peaking at superficial cortical depths. These findings not only corroborate the existing knowledge of DMN’s hierarchical organization but also offer valuable insights into the information flow direction within this network. Existing research on the DMN using dynamic causal modeling has found that the PCC sends out driven input, while the mPFC plays a regulatory role on the DMN network (Davey et al., 2016). This aligns with our finding as shown inFigure S2, where the mPFC is at the highest hierarchy level within the DMN, outputting feedback to, and receives feedforward inputs from other DMN nodes. In contrast, PCC and ANG send feedforward input to mPFC and receive feedback from mPFC.

#### 4.2.2 Concordance of connectivity- and activity-based fMRI laminar profile in somatomotor network

In the somatomotor network, we identified layer-specific connectivity patterns that align with previous layer fMRI studies focused on tasks (Chai et al., 2020;L. Huber, Handwerker, et al., 2017) and resting state (L. Huber, Handwerker, et al., 2017). Our findings revealed that the somatosensory areas (BA1 and BA3b) had peak connectivity strength at middle cortical depths, while the motor cortex (BA4a and BA4p) exhibited a distinctive double-bump laminar profile. This double-bump feature has been seen from previous activity-based and resting-state layer fMRI studies in the same motor area. When the subject is doing the finger tapping task, M1 area is activated more strongly in superficial and deep layers (Chai et al., 2020;L. Huber, Handwerker, et al., 2017). In a prior laminar resting-state fMRI study using VASO contrast, it has also been shown that M1 among the somatomotor network has a stronger connectivity not only in superficial layers but also a relatively stronger secondary peak in deeper layers (L. Huber, Handwerker, et al., 2017). This was validated in our study using layer fMRI connectivity mapping during both resting-state and movie-watching scenarios. In addition, our finding about the FCS in somatosensory areas (BA1 and BA3b) peaking at middle cortical depths is also consistent with the laminar effect of this area receiving sensory input (Yang et al., 2021;Yu et al., 2019,^2022^).

#### 4.2.3 Visual network: transition of dominance between feedforward and feedback across hierarchical levels

Our analysis of the visual network unveiled a shift in laminar profiles depending on the hierarchical level of the seed regions, thereby providing new insights into visual information processing. In a well-organized hierarchical system like the visual cortex (Lawrence et al., 2017;Markov et al., 2014;Self et al., 2017;Shipp, 2005), feedforward connections transmit information from lower- to higher-level areas, while feedback connections relay information from higher- to lower-order areas. By examining the laminar differences in connectivity between areas, we can determine the types of feedforward or feedback connections present, and thus build a hierarchy order of different brain areas. In previous layer-fMRI connectivity study using VASO, after assuming the layer profiles of feedforward and feedback connections, it has been shown that the cluster distribution of feed-forward and feedback dominance shifts according to the seed regions along the visual processing stream (L. Huber et al., 2021). In our study, we validated this at group level using a model-free k-means analysis. As the seeds migrate across LGN, V1, V2, and V3, the visual areas with a hierarchy level lower than the seed region become feedback-driven, showing peak correlation in superficial cortical depth, while the visual areas with a hierarchy level higher than the seed region become feedforward-driven, exhibiting peak correlation in middle layers (Fig. 6). This pattern is clearer when we regress out the mean visual response prior to the connectivity analysis. If we utilize the original time course without regressing out the visual response, the connectivity laminar profiles for different seeds maintain consistent, but their difference diminish (Fig. S5B). This could be attributed to the synchronized visual response to movie-watching, which renders the time course and laminar profile in visual cortex more homogeneous, thereby decreasing the difference in connectivity laminar profile from different seeds.

In the resting-state dataset, we did not observe equivalent laminar patterns in the visual cortex’s surface maps. However, a similar laminar profile emerged when we averaged the data across all vertices within each ROI, as shown inFigure S5. It is noteworthy that at the voxel/vertex level, the laminar signal appeared considerably noisier. Additionally, the number of resting-state sessions included in this study was half that of the movie-watching dataset, potentially contributing to the absence of a similar laminar pattern map in the visual cortex. Even within the somatomotor network (Fig. 5), the results, while similar, display noticeably more noise in the resting-state condition compared to the movie-watching data. This could suggest that the movie-watching condition in this study provides a more stable and distinct signal conductive to detecting laminar connectivity patterns.

#### 4.2.4 Prefrontal cortex: a central hub processing bottom-up information from the whole brain

The laminar analysis of the global hubness map revealed that the cluster with the maximum hubness at middle cortical depths is predominantly located in the prefrontal cortex (PFC). This finding suggests that the PFC acts as a high-level role within the brain’s global hierarchy, coordinating and integrating information from various regions, thus reinforcing the concept that the PFC serves as a critical hub in the brain’s functional network.

### 4.3 Considerations and limitations

#### 4.3.1 Limitations in the acquisition rate of near-whole-brain layer fMRI

While the minimum repetition time of BOLD-fMRI is mainly constrained by gradient performance and achievable acceleration with parallel imaging, non-BOLD fMRI, like VAPER-fMRI used in our study, encounters additional limitations. These stem from the slower generation of intravascular contrast and interleaved acquisition for BOLD correction, issues that are common to VAPER (Chai et al., 2020), ASL (L. Huber, Uludağ, et al., 2017), and VASO (L. Huber et al., 2014;L. Huber, Ivanov, et al., 2018;L. Huber, Tse, et al., 2018) fMRI. The near-whole-brain layer-specific VAPER-fMRI sequence in this study has been optimized for the temporal frequency window of >12 s. This acquisition rate is adequate for detection of all those common networks in both resting-state and movie-watching scenarios, as shown inFigure 3. Considering the hemodynamic delay of the vascular response, conventional resting-state fMRI is typically tuned to signal fluctuations within a time frame of 6–10 seconds, implying that fMRI is usually only sensitive to a narrow frequency window in a broad spectrum of neural fluctuations. Since resting-state fMRI fluctuations follow the pattern of scale free dynamics (He, 2011), focusing on this frequency window is expected to be largely representative of functional connections across various temporal scales. However, it is essential to note that the lengthy TR of the near-whole-brain VAPER-fMRI in this work captures only a small frequency window of a wide spectrum of neural fluctuations. Future layer-fMRI connectivity studies incorporating faster acquisition methods will be important for confirming this temporal invariance.

#### 4.3.2 Spatial resolution constraints

Typical sub-millimeter fMRI resolution of 0.8 mm at 7 T is insufficient to resolve individual histological layers, given that the total thickness of the gray matter ribbon ranges between 1.4 and 4.5 mm (Fischl & Dale, 2000). However, this level of resolution does allow for the discernment of differences across cortical depth, which can be indicative of laminar contributions on a coarser scale. Currently, the objective of human laminar fMRI studies is to distinguish the relative superficial, middle, or deep layers of the neocortex’s gray matter. Because data blurring from preprocessing and residual BOLD contamination may further degrade the effective resolution, the interpretation of laminar fMRI results in thinner cortical regions like the visual cortex—with an approximate cortical thickness of 2 mm—may be limited to only superficial and middle-deep layers (Fig. 6). This work utilized interleaved acquisition, with relatively small acceleration (3 x 2) and segmentation (2 segments) factors for shot-selective CAIPI techniques, achieving a 0.8 mm isotropic spatial resolution and layer-specific functional VAPER contrast with a reasonable effective TR of 12.164 seconds. Nonetheless, further improvement in spatiotemporal resolution is possible by means of higher acceleration (Pais-Roldán et al., 2023;Yun et al., 2022), more segmentation (Stirnberg & Stöcker, 2020), and faster gradient switching like using head-only gradient coil (Feinberg et al., 2018).

#### 4.3.3 Vascular bias in non-BOLD contrast

While the VAPER technique demonstrates a substantial reduction in superficial signal bias compared to BOLD, as evidenced inFigure 1F, it is not entirely immune to layer-unspecific vascular bias. Firstly, the control and blood-nulled images of VAPER acquisition are collected in an interleaved fashion. BOLD correction is inherently based on the underlying assumption that the BOLD contamination is not changing significantly between consecutive time points (Chai et al., 2020). In this study, due to the high resolution and near-whole-brain acquisition, the volume TR is extended during which BOLD signal could fluctuate to some degree. This causes sub-optimal BOLD correction, with a certain level of BOLD contribution in VAPER time series. Secondly, the larger vascular density of arterioles and microvessels in the superficial and middle layers may result in higher signal changes compared to the deep layers (L. Huber et al., 2015;L. Huber, Ivanov, et al., 2018). Those effects are not expected to cause a dominated signal bias toward cortical surface as in BOLD, which has already been demonstrated in activity-based layer fMRI response as shown inFigure 1F; however, it is still observable and may amplify the superficial part of the laminar profile. Due to these combined factors along with in-sufficient resolution, the potential secondary peak in deep layers (Fig. 5andFig. S2) of this study is mostly undetectable and blurred by the main laminar trend.

To mitigate the residual vascular bias in VAPER imaging, one strategy could involve decreasing the volume TR. This could be achieved by adopting advanced acceleration techniques in future studies, such as low-rank reconstruction (R. Jin et al., 2023,^2024^;Liang, 2007) to expedite the acquisition, thereby minimizing BOLD signal variations between consecutive time points and enhancing BOLD correction accuracy. Additionally, by comparing laminar profiles across varying conditions expected to exhibit identical vascular biases but distinct laminar patterns (Chai, Liu, et al., 2021), we can more accurately differentiate true neuroscientific phenomena from the confounding effects of residual macrovascular bias.

## 5 Conclusions

This study adopted a novel layer-fMRI sequence tool to map laminar functional connectivity in a 7 T scanner with near-whole-brain coverage in healthy subjects. By offering insights into the laminar profiles of global hubness and different nodes within the DMN, somatomotor, and visual networks, we have identified distinct connectivity patterns that align with known hierarchical structures. These findings not only confirm the feasibility of using VAPER for layer fMRI connectivity research but also illuminate new aspects of the brain’s functional organization. Given that disturbances in cortical networks are often associated with psychiatric disorders, our study’s implications could extend to improving our understanding and treatment of such conditions. We believe that our findings pave the way for more detailed and nuanced studies of functional brain connectivity.

## Supplementary Material

Supplementary Material

## Data Availability

Datasets and analysis code are available from the OSF repository (DOI:https://doi.org/10.17605/OSF.IO/H9U64). Analysis code is also available inhttps://github.com/yuhuichai/LayConn. The analysis pipeline uses publicly available software packages, including FreeSurfer (https://freesurfer.net/), AFNI+SUMA (https://afni.nimh.nih.gov/), SPM (https://www.fil.ion.ucl.ac.uk/spm/), ANTs (http://stnava.github.io/ANTs/), and LAYNII (https://github.com/layerfMRI/LAYNII).
