# Supplementary material for: Unlocking near-whole-brain, layer-specific functional connectivity with 3D VAPER fMRI
Source: Imaging Neurosci (Camb). 2024 Apr 18;2:imag-2-00140. doi: 10.1162/imag_a_00140 (PMC12247610; doi:10.1162/imag_a_00140)
Supplement: Supplementary Material [file imag_a_00140-supp.pdf]

SUPPLEMENTARY MATERIALS

Dice similarity coefficient for k-means parcellations (2 clusters) across different datasets

The Dice similarity coefficient quantifies the degree of overlap in parcellation outcomes between different datasets. Given two datasets, Dice’s coefficient computes the ratio of the number of vertices that belongs to the same cluster (either k = 1 or k = 2, where k = 1 corresponds to laminar profile peaking in superficial layers and k = 2 corresponds to laminar profiles peaking in middle layers) to the total number of vertices within that specific network area. Dice's coefficient results in numbers between zero and one, where one corresponds to perfect correspondence between matrices, and zero corresponds to no similarity. This coefficient serves as a meaningful metric for evaluating the similarity of parcellation results across different datasets.

Table S1. Number of scan sessions conducted for each experiment condition in each subject. Each scan session had 2-3 functional runs for resting-state or 4-5 functional runs for movie-watching, spanning a total duration of 2 hours. Different sessions within the same subject occurred on different days. For example, subject 2 participated in one session of the movie-watching experiment and three sessions of the resting-state experiment, resulting in a total scanning time of 8 hours.

	Subj 1	Subj 2	Subj 3	Subj 4	Subj 5	Subj 6	Subj 7	Subj 8	Subj 9	Subj 10	Subj 11	Subj 12
Movie-watching (number of sessions)	1	1	0	5	5	1	5	1	5	0	0	0
Resting-state (number of sessions)	1	3	2	1	1	0	0	0	0	1	2	1

Table S2. Summary of Dice similarity coefficients and t-test statistics.

(A) Dice similarity coefficients for each network between movie-watching and resting-state datasets, between two subsets comprising the first and second halves individuals, between sub-groups involving only half individuals and the whole-group encompassing all individuals.

	Movie-watching vs. resting state	Movie-watching: 1st subset vs. 2nd subset	Movie-watching: half individuals vs. all individuals	Resting-state: 1st subset vs. 2nd subset	Resting-state: half individuals vs. all
DMN	0.80	0.74 ± 0.15	0.75 ± 0.03	0.70 ± 0.16	0.71 ± 0.03
Somatomotor	0.65	0.74 ± 0.16	0.69 ± 0.05	0.70 ± 0.16	0.72 ± 0.05
Visual		0.63 ± 0.19	0.63 ± 0.03		
Global hubness	0.78	0.75 ± 0.14	0.76 ± 0.04	0.71 ± 0.15	0.72 ± 0.05

(B) Dice similarity coefficients for random patterns pairs as null hypothesis. As we already defined the mask for each network and each vertex within the network was classified as either 1 or 2 (corresponding to laminar profiles peaking in superficial layers or middle layers, respectively), the Dice similarity coefficients have a mean value of 0.5 for random pattern pairs in each network.

	Random pattern 1 vs. random pattern 2	k-means parcellation from movie-watching dataset vs. random pattern	k-means parcellation from resting-state dataset vs. random pattern
DMN	0.5 ± 0.002	0.5 ± 0.001	0.5 ± 0.001
Somatomotor	0.5 ± 0.001	0.5 ± 0.001	0.5 ± 0.001
Visual	0.5 ± 0.002	0.5 ± 0.001	
Global hubness	0.5 ± 0.001	0.5 ± 0.001	0.5 ± 0.001

(c) p-values for the two-sample t-test between Dice's coefficients obtained from actual datasets vs. those derived from the null hypothesis

	Movie-watching	Resting-state
DMN	0	3.6415e-14
Somatomotor	0	3.8414e-14
Visual	4.2797e-06	
Global hubness	0	6.6613e-16

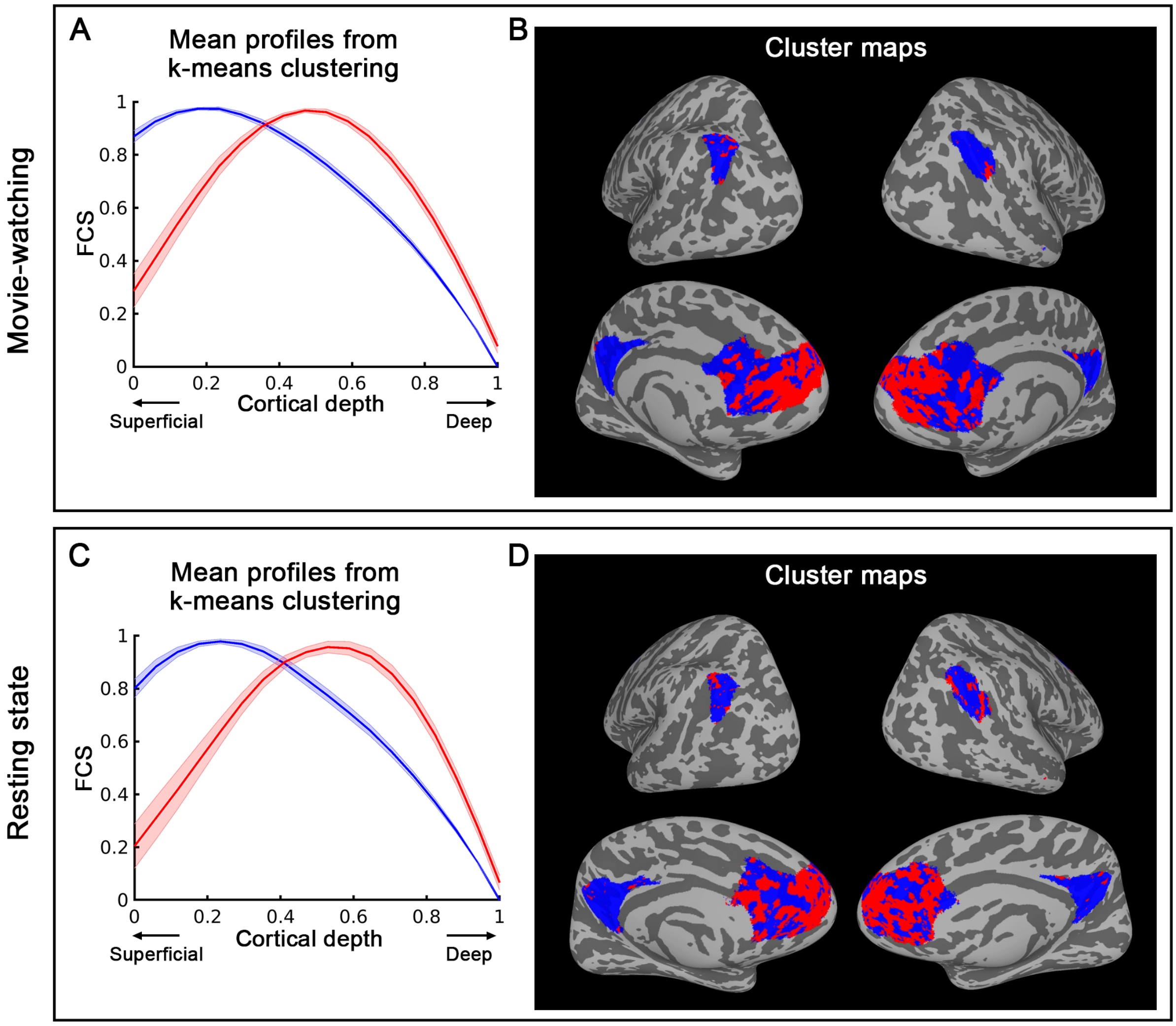

Figure S1. Laminar profile based parcellation of default mode network (DMN) using k-means clustering at the group level (N = 24 for movie-watching and N = 12 for resting state). The upper panel showcases the results from the movie-watching experiment, while the lower panel presents results from the resting-state experiment. The DMN network region was defined using the apriori DMN from Yeo atlas (Thomas Yeo et al., 2011). k = 2 was employed, and the mean FCS for each cluster was plotted as a function of cortical depth, as shown in (A) and (C). The shaded areas represent ± SEM (standard error of the mean) across sessions. In (B) and (D), red regions represent FCS peaking at middle cortical depths, while the blue regions indicate FCS peaking at superficial cortical depths.

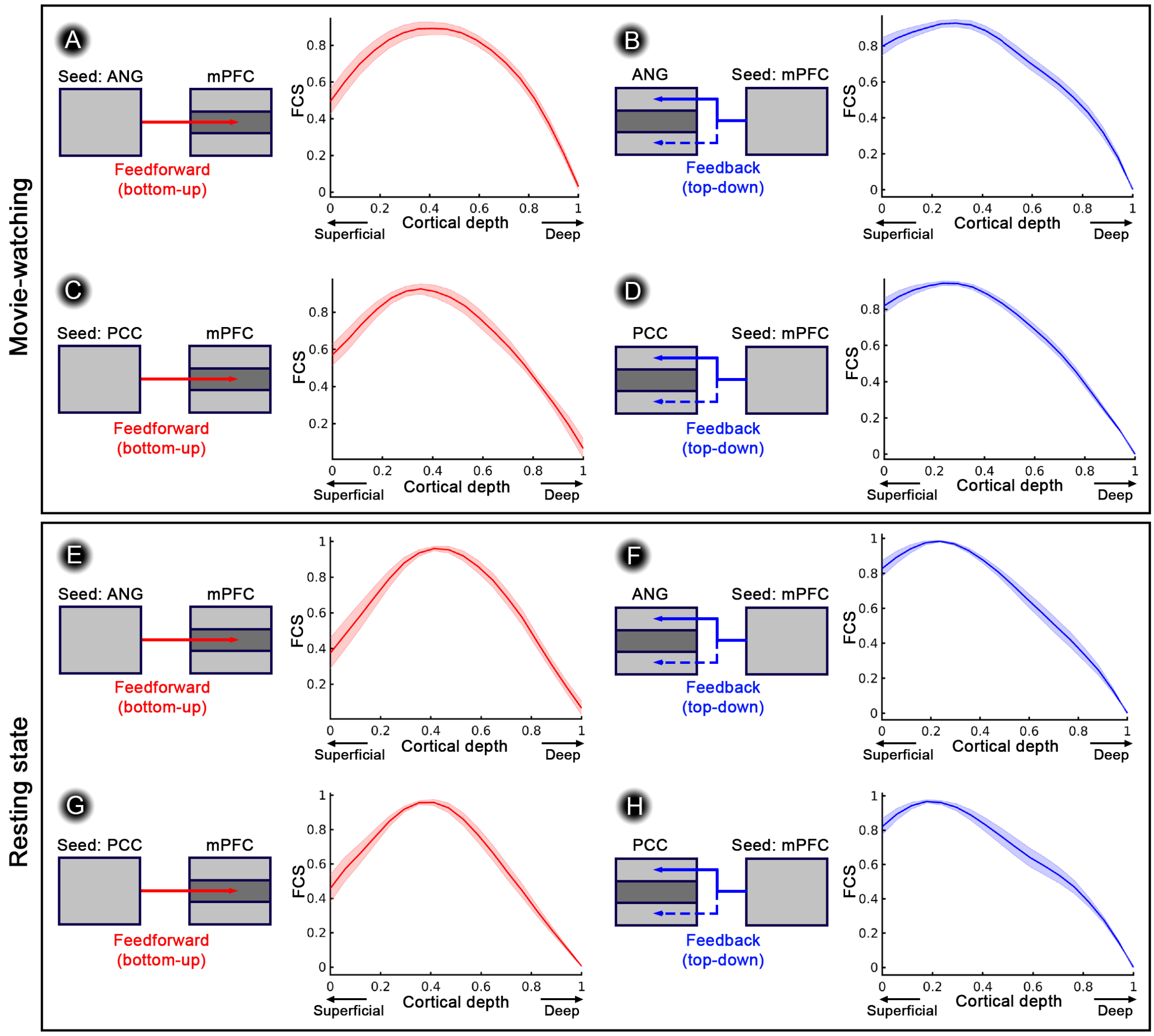

Figure S2. Laminar profile of seed-based FCS within default mode network (DMN). Each sub-network node was used as the seed for connectivity analysis. The results of both the movie-watching and resting-state experiments are displayed in the upper and lower panels, respectively. (A), (C), (E) and (G) depict the seed ROIs in the angular gyrus (ANG) or posterior cingulate cortex (PCC) and their correlations with different cortical depths in mPFC. (B), (D), (F) and (H) show the seed ROIs in mPFC and their correlation with different cortical depths in the ANG or PCC. The curve plots depicting the laminar profiles were averaged across all individuals (N = 24 for movie-watching and N = 12 for resting state), with the shaded areas representing ± SEM.

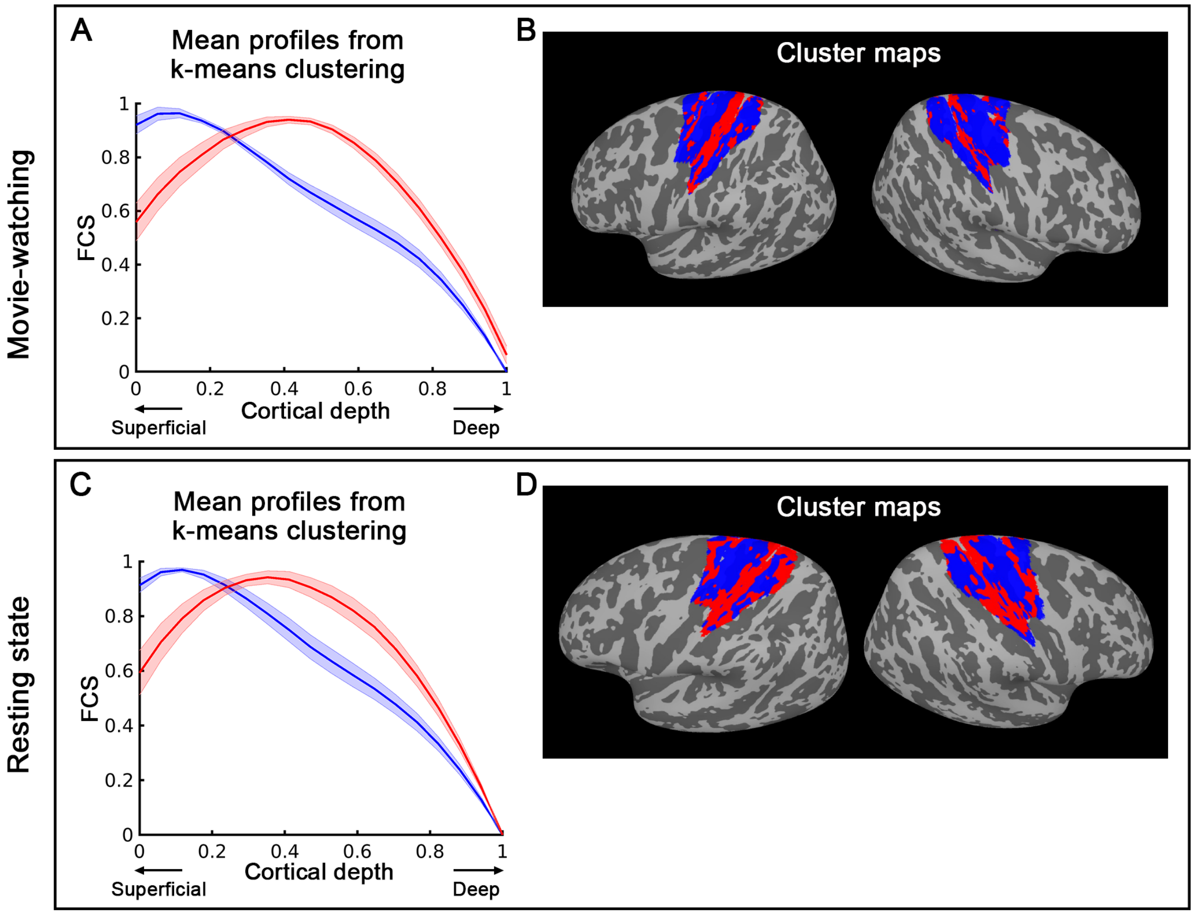

Figure S3. Laminar profile based parcellation of somatomotor network using k-means clustering at the group level (N = 24 for movie-watching and N = 12 for resting state). The upper panel displays the results from the movie-watching experiment, while the lower panel presents results from the resting-state experiment. The somatomotor region used the apriori somatomotor network from Yeo atlas (Thomas Yeo et al., 2011). k = 2 was employed, and the mean FCS for each cluster was plotted as a function of cortical depth, as shown in (A) and (C). The shaded areas represent ± SEM across sessions. In (B) and (D), red regions have a laminar profile of FCS peaking at middle cortical depths. Conversely, the blue regions indicate a laminar profile peaking at superficial and slightly at deep cortical depths.

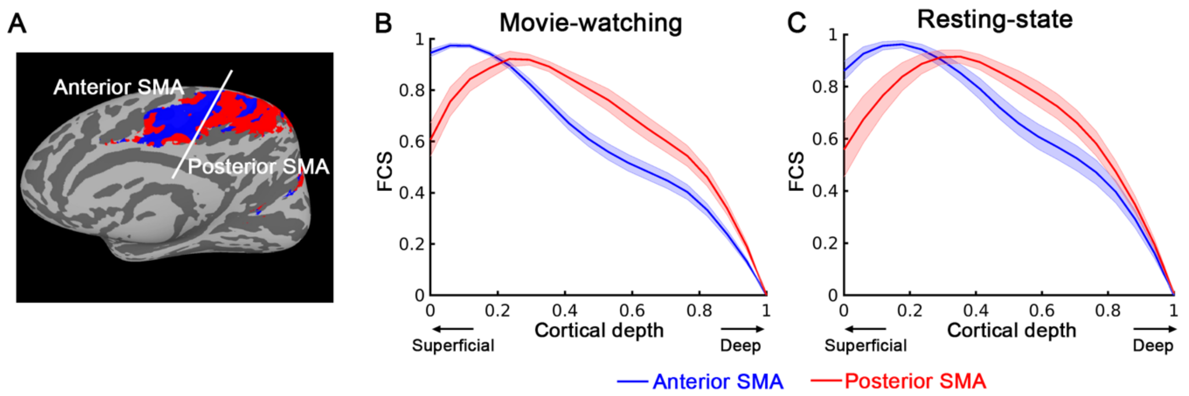

Figure S4. Laminar FCS profiles in anterior and posterior Supplementary Motor Area (SMA) within somatomotor network. (A) The delineation between anterior and posterior SMA (Lima et al., 2016) is indicated by the white line on the k-means clustering map of the somatomotor network. (B) and (C) depict the mean FCS laminar profiles for each SMA subregion during movie-watching and resting-state experiment, respectively. Notable differences in the FCS laminar profiles the two subregions may reflect their different function roles.

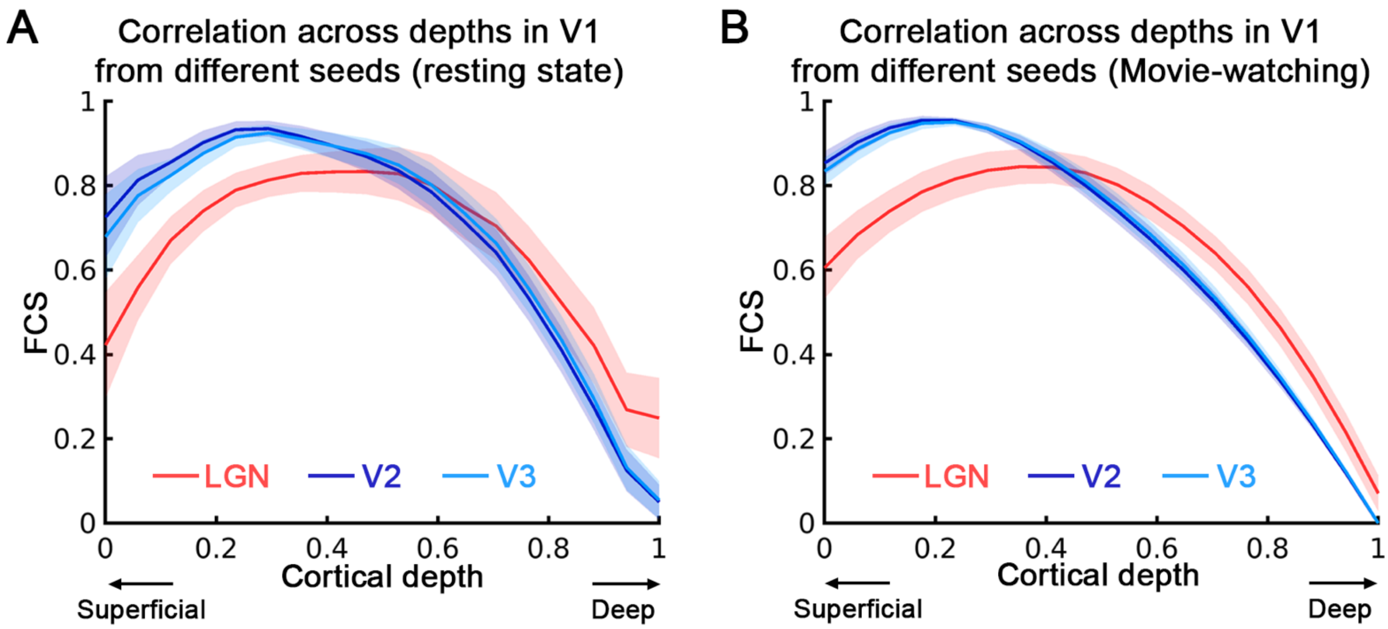

Figure S5. The mean layer profiles of FCS in V1 with seeds in LGN, V2 and V3 for (A) resting state (N = 12) and (B) movie-watching (N = 24). The time series used for connectivity analysis here have not been regressed out against any mean visual response. When using the seed in LGN, V1 exhibits peak FCS at middle cortical depths. However, with seeds in V2 and V3, the peak FCS in V1 shift towards the superficial cortical depths. The shaded areas represent ± SEM across sessions.
